# Photonics-Aided 20 m Wireless Transmission of 56-GBaud OFDM Signals at 138 GHz in the D-Band for 6G Applications

**DOI:** 10.3390/s26103250

**Published:** 2026-05-20

**Authors:** Hanyu Zhang, Zhongxiao Pei, Qinyi Zhang, Yifan Chen, Jianjun Yu

**Affiliations:** State Key Laboratory of ASIC and System, Key Laboratory for Information Science of Electromagnetic Waves (MoE), School of Information Science and Technology, Fudan University, Shanghai 200433, China; 24210720317@m.fudan.edu.cn (H.Z.);

**Keywords:** D-band wireless transmission, optical heterodyne generation, homodyne reception, indoor wireless links, OFDM-QPSK, sub-THz communications, 6G wireless access, frequency-selective distortion

## Abstract

To meet the demand for high-capacity indoor wireless access in future 6G systems, we propose and experimentally demonstrate a photonics-aided D-band wireless transmission scheme operating at 138 GHz. At the transmitter, two external-cavity lasers together with an I/Q modulator are used to generate a modulated D-band carrier. At the receiver, homodyne down-conversion is employed to directly recover the received signal to baseband, thereby relaxing the requirements on ultra-wideband analog components and high-speed sampling hardware. A 20 m indoor line-of-sight wireless link is established to transmit a 56-Gbaud-rate OFDM-QPSK signal. The transmitted and received spectra, received constellations and bit-error-rate (BER) performance are functions of optical power at different symbol rates, and the channel amplitude and phase responses are systematically analyzed. The results show that broadband D-band signal generation, transmission, and recovery can be stably achieved in the proposed system. After receiver-side digital signal processing (DSP), clear QPSK constellations are obtained. BER measurements reveal an optimal optical-power operating range, and the 32-GBaud OFDM signal outperforms the 56-Gbaud-rate signal because its narrower occupied bandwidth makes it less sensitive to frequency-selective distortion. For 56-Gbaud-rate OFDM transmission, the BER approaches the 20% low-density parity-check forward-error-correction threshold at an optical power of approximately −1 dBm. Further analysis indicates that the current link performance is mainly limited by frequency-selective amplitude and phase distortions under bandwidth-constrained conditions, together with slight nonlinear effects at high power. These results verify the feasibility of a photonics-aided D-band wireless architecture with homodyne reception for medium-range, high-symbol-rate indoor transmission and provide an experimental basis for future 6G sub-THz wireless links.

## 1. Introduction

With the rapid growth of ultra-high-definition video, immersive interaction, cloud-edge collaborative computing, and intelligent terminal services, future sixth-generation (6G) wireless networks are expected to provide substantially higher throughput, denser connectivity, and lower latency than current systems [1,2,3,4]. Meeting these requirements with conventional microwave and millimeter-wave spectrum alone is increasingly difficult because the available contiguous bandwidth is inherently limited. Consequently, wireless transmission above 100 GHz, especially in the sub-terahertz and terahertz regimes, has emerged as a key candidate for future high-capacity short-range access and backhaul owing to its abundant spectrum resources and potential for extremely high data rates [5,6,7,8,9,10].

Among the candidate frequency windows above 100 GHz, the D-band (110–170 GHz) offers an attractive compromise among available bandwidth, propagation characteristics, device maturity, and implementation feasibility. Compared with carrier frequencies far above 300 GHz, D-band systems are generally more practical for indoor wireless access and short-/medium-range backhaul scenarios [11,12,13]. In parallel, photonics-aided carrier generation has become an important enabling approach for sub-THz systems because optical heterodyne beating between two optical tones can provide wide frequency tunability, large modulation bandwidth, and natural compatibility with fiber distribution networks, thereby supporting seamless fiber–wireless convergence [14,15,16,17,18].

Recent photonics-aided D-band and sub-THz wireless demonstrations have confirmed the feasibility of high-capacity transmission above 100 GHz. Previous studies have mainly advanced the field along three directions. The first direction is to increase the data rate by using high-order modulation, probabilistic shaping, polarization or spatial multiplexing, and advanced DSP. For example, PS-16QAM-OFDM transmission at 350 GHz has achieved beyond-100-Gbit/s-class wireless/RoF transmission over a 26.8 m wireless link [19,20,21], while more recent >300-GHz MIMO or dual-polarization systems have further increased the achievable capacity by using polarization multiplexing, MIMO equalization, and multidimensional nonlinear compensation [22,23,24]. The second direction is to extend the wireless reach by using high-gain antennas or lenses, diversity reception, THz amplifiers, LNAs, or SIMO/MRC processing, as demonstrated in long-distance D-band and THz links. The third direction is to improve robustness against phase noise, dispersion, multipath fading, and synchronization errors through self-heterodyne reception, intermediate-frequency reception, IM/DD architectures, neural-network equalizers, or dedicated sampling-frequency offset compensation [25,26,27,28,29].

Although these works have achieved impressive data rates or transmission distances, many of them rely on relatively complex front-end configurations, intermediate-frequency or self-heterodyne reception, high-gain diversity/multiplexing hardware, or computationally intensive equalization. In contrast, for indoor D-band access links, receiver simplicity, direct baseband recovery, and stable high-symbol-rate operation are also important design considerations [30,31,32]. Therefore, a more compact photonics-aided D-band architecture with homodyne/zero-IF reception is worth investigating experimentally, especially for medium-range indoor line-of-sight transmission [33].

In this work, we propose and experimentally demonstrate a photonics-aided 138-GHz D-band wireless transmission system using homodyne down-conversion at the receiver. The proposed receiver directly converts the received D-band signal to baseband, avoiding a broadband intermediate-frequency signal chain and relaxing the requirements on ultra-wideband RF/IF analog components. Based on this architecture, a 56-GBaud OFDM-QPSK signal is transmitted over a 20 m indoor line-of-sight wireless link. Compared with prior works that mainly emphasize record capacity, long-distance transmission, MIMO/polarization multiplexing, or advanced nonlinear/NN-based equalization, this work focuses on the experimentally validated trade-off among high OFDM symbol rate, moderate indoor reach, and reduced receiver complexity in the D-band.

For clarity, Table 1 summarizes representative OFDM wireless transmission demonstrations at the D-band and above reported in the last decade, with emphasis on carrier frequency, signal format, reported rate, wireless distance, and receiver architecture.

To address this issue, this work proposes and experimentally demonstrates a photonics-aided D-band wireless transmission scheme operating at 138 GHz for 6G-oriented indoor links. At the transmitter, dual external-cavity lasers together with an IQ modulator are employed to generate a modulated D-band carrier via optical heterodyne beating. At the receiver, homodyne down-conversion directly translates the received signal to baseband, thereby relaxing the bandwidth requirements on ultra-wideband analog components and high-speed sampling hardware. Based on this architecture, a 56-GBaud OFDM-QPSK signal is successfully transmitted over a 20 m indoor line-of-sight wireless link. The transmitted and received spectra, recovered constellations, BER performance under different optical power conditions, and channel amplitude/phase responses are systematically characterized. The experimental results verify a practically relevant trade-off among high symbol rate, moderate indoor reach, and reduced receiver complexity, and provide experimental support for future D-band sub-THz indoor access systems.

The main contributions of this work are summarized as follows:

(1) A photonics-aided 138-GHz D-band OFDM wireless transmission architecture with homodyne/zero-IF reception is experimentally demonstrated. Different from IF- or self-heterodyne-based sub-THz receivers, the proposed receiver directly down-converts the received D-band signal to baseband, thereby simplifying the receiver-side frequency-conversion chain.
(2) A 56-GBaud OFDM-QPSK signal is transmitted over a 20 m indoor line-of-sight wireless link. The corresponding net payload rate is 81.16 Gbit/s when the periodic training-symbol overhead is excluded, and 67.63 Gbit/s when the experimentally used one-training-symbol-per-five-data-symbol overhead is included.
(3) In contrast to prior photonics-aided D-band/sub-THz demonstrations that mainly emphasize record capacity, long-distance transmission, high-order modulation, MIMO/polarization multiplexing, or complex NN/nonlinear equalization, this work investigates a practically relevant indoor operating point that balances high OFDM symbol rate, moderate wireless reach, and reduced receiver complexity.
(4) System performance is systematically characterized through optical-power-dependent BER, transmitted and received spectra, recovered constellations, and channel amplitude/phase responses. The results identify bandwidth-constrained frequency-selective amplitude/phase distortion and mild high-power nonlinearity as the dominant limitations of the present homodyne D-band link.

## 2. Experimental System and Configuration

**Figure 1:**
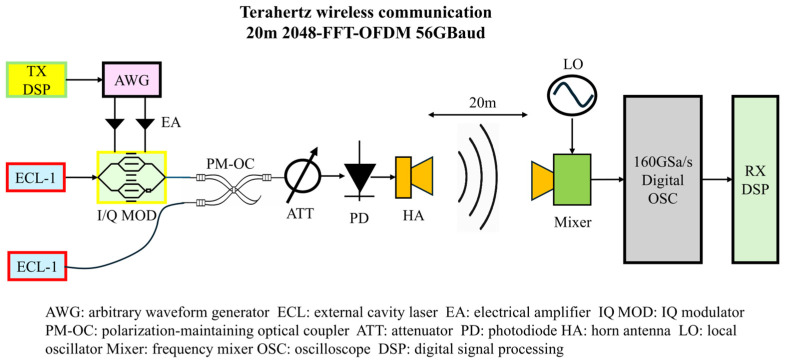
Experimental setup.

**Figure 2:**
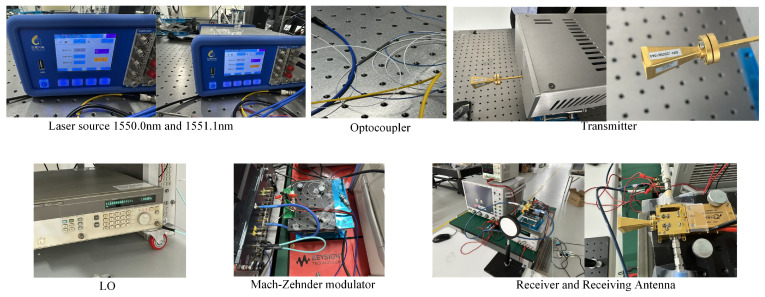
Pictures of the experimental setup.

To validate the proposed D-band high-speed wireless transmission photonics-aided D-band wireless transmission scheme, an experimental platform was constructed comprising a photonics-aided transmitter, a 20 m indoor line-of-sight wireless link, and a homodyne receiver; the system block diagram and photographs of the experimental setup are shown in Figure 1 and Figure 2, respectively. The entire system follows a technical workflow involving: offline DSP for baseband waveform generation; an Arbitrary Waveform Generator (AWG) for electrical driving signal output; an I/Q Mach-Zehnder Modulator (IQ-MZM) for optical-domain modulation; optical heterodyne generation of a 138 GHz RF carrier; free-space transmission; homodyne down-conversion reception; digital oscilloscope sampling; and offline DSP for data recovery. The transmitted signal in the experiment was 56-GBaud OFDM-QPSK and 32-GBaud OFDM-QPSK. The OFDM parameters used in the experiment are summarized in Table 2.

The net payload rate of the 56-GBaud OFDM-QPSK signal is calculated according to the number of data-bearing subcarriers and the OFDM symbol overhead. In this experiment, the FFT size is 2048, among which 1600 subcarriers are used for data transmission. The cyclic prefix and cyclic suffix lengths are 128 and 32 samples, respectively. Therefore, excluding the periodic training-symbol overhead, the net payload rate can be calculated as

(1) $$R_{\mathrm{net}}=R_{s}\;\log_{2}(M)\frac{N_{\mathrm{data}}}{N_{\mathrm{FFT}}+N_{\mathrm{CP}}+N_{\mathrm{CS}}}$$

where Rs = 56 GBaud is the signal baud rate, M = 4 is the QPSK modulation order, $N_{\mathrm{data}}=1600,\;N_{\mathrm{FFT}}=2048,\;N_{\mathrm{CP}}=128,\;\mathrm{and}\;N_{\mathrm{CS}}=32$. Thus

(2) $$R_{\mathrm{net}}=56\times 2\times \frac{1600}{2048+128+32}=81.16\;\mathrm{Gbit}/s$$

In the BER measurement, one training OFDM symbol is inserted after every five data OFDM symbols for synchronization. If this training-symbol overhead is strictly included, the effective throughput becomes

(3) $$R_{\mathrm{eff}}=R_{\mathrm{net}}\frac{N_{D}}{N_{D}+N_{T}}=81.16\times \frac{5}{5+1}=67.63\;\mathrm{Gbit}/s,$$

It should be noted that the training symbol is mainly used for frame synchronization in the current experiment. For a quasi-static indoor line-of-sight D-band link, one training symbol can be used to support a larger number of subsequent data frames after reliable synchronization is achieved. Therefore, by increasing the training interval, the training overhead can be reduced, and the achievable net payload rate can asymptotically approach 81.16 Gbit/s.

### 2.1. Transmitter Implementation

At the transmitter end, an offline DSP first performs pseudo-random bit sequence generation, QPSK mapping, pilot insertion, training symbol construction, and OFDM frame assembly to produce complex baseband I/Q waveforms. Subsequently, the generated digital waveforms are loaded into an AWG, which outputs two analog electrical drive signals. These signals are then amplified by Electrical Amplifiers (EAs) and applied to an IQ-MZM to achieve complex modulation of the optical carrier. In the experiment, External Cavity Lasers (ECLs) serve as light sources, with their center wavelengths set to 1550.0 nm and 1551.1 nm, respectively; one serves as the optical carrier input to the IQ-MZM, while the other acts as an unmodulated continuous-wave optical signal. The two optical signals are combined via a polarization-maintaining optical coupler (PM-OC) and directed into a high-speed Photodetector (PD—specifically, UTC-PD), where a modulated 138 GHz D-band signal is generated via optical heterodyne beating in the UTC-PD. To ensure efficient beating and stable operation of the device within its linear region, an Adjustable Optical Attenuator (ATT) is placed prior to the PD to optimize the combined optical power. The resulting RF signal is then radiated into free space via a horn antenna.

The relevant device parameters are shown in Table 3.

It should be noted that the linewidths of the two ECLs are important parameters for the quality of the generated D-band carrier. In optical heterodyne generation, the RF carrier phase is determined by the relative phase between the two optical tones. Therefore, the generated RF phase noise is related to the combined linewidth of the two ECLs. Since both ECLs used in this experiment have linewidths below 100 kHz, the heterodyne beat linewidth is estimated to be below 200 kHz under the assumption of independent laser phase noise. This value is much smaller than the subcarrier spacing of the 56-GBaud OFDM signal, and the resulting phase impairment can be mainly treated as carrier-frequency offset and common phase error in the receiver DSP.

### 2.2. Wireless Link and Receiver Implementation

The transmitter and receiver are positioned face-to-face, utilizing directional horn antennas to establish a 20 m indoor wireless link. At the receiver, the horn antenna first captures the modulated D-band signal and feeds it into a frequency mixer. To facilitate homodyne reception, a local oscillator signal is generated by an 11.5-GHz microwave source and then multiplied by a factor of 12 to provide a nominal 138-GHz LO. The LO frequency is nominally aligned with the received D-band carrier, while the residual carrier-frequency mismatch is estimated and compensated in the receiver-side DSP. The down-converted baseband waveform is sampled and digitized by a digital oscilloscope featuring an 80 GHz bandwidth and a sampling rate of 160 GSa/s. The resulting offline data is then fed into the receiver’s DSP unit to execute the various signal-processing tasks outlined in Section 3—including symbol synchronization, frequency offset estimation and compensation, channel estimation, equalization, and phase recovery. Compared to schemes involving direct broadband sampling of the D-band RF signal, this homodyne receiver effectively mitigates the receiver’s demands for ultra-high-frequency analog bandwidth and high-speed electronic front-ends, thereby facilitating the practical engineering implementation of current D-band experimental platforms.

### 2.3. Experimental Platform Description

Figure 1 and Figure 2 illustrate the key physical components of the experimental platform, comprising a dual-wavelength laser source, a polarization-maintaining optical coupler, a D-band transmitting front-end, a local oscillator source, an IQ-MZM, a receiving front-end, and a receiving antenna. During the experiment, efficient optical heterodyne beating in the UTC-PD and the stability of the 20 m line-of-sight link were ensured by jointly adjusting the output power of the ECLs, the operating point of the variable optical attenuator, and the spatial alignment of the transmitting and receiving antennas. Simultaneously, by optimizing the local oscillator power and the mixing operating point, a high effective signal-to-noise ratio was achieved for the down-converted baseband signal within the full-scale range of the digital oscilloscope. This experimental configuration established a unified hardware foundation for the subsequent testing and analysis of the transmitting and receiving spectra, received constellation diagrams, BER, and channel frequency responses.

## 3. Receiver-Side DSP for OFDM Signal Recovery

To recover the OFDM baseband data after 20 m D-band wireless transmission, offline receiver-side DSP was performed on the waveform acquired by the digital storage oscilloscope (DSO). Unlike direct OFDM demodulation at the oscilloscope sampling rate, the received waveform was first subjected to DC-offset removal, amplitude normalization, and rational resampling before entering the subsequent OFDM demodulation process. The DSP chain used in this work includes DC-offset removal, amplitude normalization, rational resampling, frame synchronization, frequency offset estimation and compensation, FFT-window extraction, least-squares (LS) channel estimation, pilot-assisted one-tap frequency-domain equalization, and residual common phase error (CPE) correction. The constellation diagrams, BER, and channel response analysis presented in Section 4 were obtained using this DSP procedure.

### 3.1. Digital Sampling and Preprocessing

After homodyne down-conversion, the receiver-side baseband waveform was acquired by the DSO at $f_{DSO}=160$ GSa/s. The offline receiver-side DSP was not performed directly at the oscilloscope sampling rate; instead, after preprocessing, the sampled waveform was resampled to the nominal OFDM sampling rate. For the 56-GBaud and 32-GBaud OFDM signals, the target DSP sampling rates were $R_{s}=56$ GSa/s and $R_{s}=32$ GSa/s, respectively. Therefore, the resampling ratios from the DSO sampling rate to the DSP sampling rate were 7/20 and 1/5, respectively.

Let $y_{DSO}\left[n\right]$ denote the discrete-time baseband waveform acquired by the DSO. The DC component is first removed as follows:

(4) $$\mu_{y}=\frac{1}{N}\sum_{n=0}^{N-1}y_{\mathrm{DSO}}[n]$$

(5) $$y_{0}[n]=y_{\mathrm{DSO}}[n]-\mu_{y}$$

where N is the number of sampling points used for offline processing. The waveform is then normalized to unit average power:

(6) $$y_{1}[n]=\frac{y_{0}[n]}{\sqrt{\frac{1}{N}\sum_{n=0}^{N-1}{\left|y_{0}[n]\right|}^{2}}}$$

After DC-offset removal and amplitude normalization, rational resampling is performed. The resampled sequence used for OFDM demodulation can be expressed as

(7) $$r[n]=R_{R_{s}/f_{\mathrm{DSO}}}\{y_{1}[n]\}$$

where $R_{R_{s}/f_{DSO}}\{\cdot \}$ denotes the resampling operation from $f_{DSO}$ to $R_{s}$. Specifically, the 56-GBaud signal corresponds to $R_{s}/f_{DSO}=7/20$, and the 32-GBaud signal corresponds to $R_{s}/f_{DSO}=1/5$. After this operation, one sample of $r\left[n\right]$ corresponds to one OFDM time-domain sample. Therefore, the subsequent symbol synchronization, frequency offset compensation, cyclic-prefix removal, FFT, channel estimation, equalization, and phase recovery are all performed on the resampled sequence $r\left[n\right]$.

Apart from the interpolation/anti-aliasing filtering inherent to the rational resampling process, no additional digital filtering was applied before OFDM demodulation. Meanwhile, the current DSP procedure did not employ separate I/Q imbalance compensation. The residual frequency-selective amplitude and phase responses jointly introduced by the transmitter, wireless link, and receiver are estimated and compensated by the pilot-assisted frequency-domain equalizer described in Section 3.4.

### 3.2. Symbol Synchronization and FFT-Window Selection

After resampling, all sample indices are defined at the target OFDM sampling rate $R_{s}$. In this experiment, each OFDM frame consists of one training OFDM symbol and five data OFDM symbols. Each OFDM symbol contains $N_{FFT}=2048$ useful samples, $N_{CP}=128$ cyclic-prefix samples, and $N_{CS}=32$ cyclic-suffix samples. Therefore, the sampling interval between the starting positions of two adjacent effective FFT windows is

(8) $$N_{\mathrm{sym}}=N_{\mathrm{FFT}}+N_{\mathrm{CP}}+N_{\mathrm{CS}}=2048+128+32=2208$$

Frame synchronization is implemented using the training OFDM symbol. The effective time-domain part of the training symbol is designed with a conjugate symmetric structure, i.e.,

(9) $$s_{\mathrm{tr}}[n]=\mathrm{conj}\left(s_{\mathrm{tr}}\left[N_{\mathrm{FFT}}-1-n\right]\right),\;0\leq n<N_{\mathrm{FFT}}$$

where $conj\left(\cdot \right)$ denotes complex conjugation, and $s_{tr}\left[n\right]$ denotes the effective time-domain part of the transmitted training symbol. Based on this conjugate symmetry property, a normalized mirror-correlation metric can be calculated on the resampled received sequence $r\left[n\right]$. For a candidate starting index d of the effective block of the training symbol, the correlation term and energy terms are first calculated as follows:

(10) $$C(d)=\sum_{n=0}^{L-1}r[d+n]r\left[d+N_{\mathrm{FFT}}-1-n\right]$$

(11) $$E_{1}(d)=\sum_{n=0}^{L-1}{\left|r[d+n]\right|}^{2}$$

(12) $$E_{2}(d)=\sum_{n=0}^{L-1}{\left|r\left[d+N_{\mathrm{FFT}}-1-n\right]\right|}^{2}$$

where $L=N_{FFT}/2$. The normalized mirror-correlation metric is defined as

(13) $$M(d)=\frac{{\left|C(d)\right|}^{2}}{E_{1}(d)E_{2}(d)}$$

The starting position of the effective part of the training OFDM symbol is determined from the correlation peak:

(14) $${\hat{d}}_{0}=\arg\max_{d}\;M(d)$$

After ${\hat{d}}_{0}$ is determined, the effective FFT window of the mth OFDM symbol within the frame is extracted as

(15) $$r_{m}[n]=r\left[{\hat{d}}_{0}+mN_{\mathrm{sym}}+n\right],\;0\leq n<N_{\mathrm{FFT}}$$

where m=0 corresponds to the training OFDM symbol, and m=1,…,5 corresponds to the five data OFDM symbols. With this window-selection method, the CP and CS samples do not enter the FFT operation. In the actual DSP procedure, the final FFT-based OFDM demodulation is performed after frequency offset compensation, as described in the next subsection.

### 3.3. Frequency Offset Estimation and Compensation

After frame synchronization, the received OFDM signal still contains residual carrier frequency offset. This frequency offset mainly arises from the frequency mismatch between the optoelectronically generated D-band carrier and the frequency-multiplied electrical local oscillator at the receiver. In the DSP implementation, the carrier frequency offset is normalized by the OFDM subcarrier spacing:

(16) $$\varepsilon =\frac{\Delta f}{\Delta f_{\mathrm{sub}}}$$

where Δf is the residual carrier frequency mismatch after homodyne down-conversion, and $\Delta f_{sub}$ is the OFDM subcarrier spacing. Because the DSP is performed after resampling to the nominal OFDM sampling rate $R_{s}$, the subcarrier spacing is

(17) $$\Delta f_{\mathrm{sub}}=\frac{R_{s}}{N_{\mathrm{FFT}}}$$

For the 56-GBaud OFDM signal, the subcarrier spacing is 56 GHz/2048 = 27.34 MHz; for the 32-GBaud OFDM signal, the subcarrier spacing is 32 GHz/2048 = 15.63 MHz.

Frequency offset compensation is performed in two steps. First, the cyclic prefix is used to estimate the fractional frequency offset within one subcarrier spacing. Let the starting index of the mth OFDM symbol including the CP be

(18) $$d_{m}={\hat{d}}_{0}-N_{\mathrm{CP}}+mN_{\mathrm{sym}}$$

The CP-based correlation term is calculated as

(19) $$P=\sum_{m=0}^{N_{\mathrm{frm}}-1}\sum_{n=0}^{N_{\mathrm{CP}}-1}r\left[d_{m}+n+N_{\mathrm{FFT}}\right]\mathrm{conj}\left(r[d_{m}+n]\right)$$

where $N_{frm}=6$ denotes the number of OFDM symbols within one frame, including one training symbol and five data symbols. The fractional frequency offset estimate is

(20) $${\hat{\varepsilon }}_{\mathrm{frac}}=\frac{1}{2\pi }\arg(P),\;-\frac{1}{2}\leq {\hat{\varepsilon }}_{\mathrm{frac}}<\frac{1}{2}$$

The CP-based estimator can obtain only the fractional part of the normalized frequency offset. Therefore, the residual integer subcarrier offset is further estimated using the known training OFDM symbol. After fractional frequency offset compensation, an FFT is performed on the training symbol, and a correlation search is conducted over candidate integer subcarrier offsets:

(21) $${\hat{k}}_{\mathrm{int}}=\arg\max_{q}\left|\sum_{k\in A}Y_{0,\mathrm{frac}}[k+q]\mathrm{conj}\left(X_{0}[k]\right)\right|$$

where $X_{0}\left[k\right]$ is the transmitted training symbol on the kth active subcarrier, $Y_{0,frac}\left[k\right]$ is the received training symbol after fractional frequency offset compensation, A denotes the active subcarrier set, and q denotes a candidate integer subcarrier offset within the preset search range. The total normalized frequency offset can be expressed as

(22) $$\hat{\varepsilon }={\hat{k}}_{\mathrm{int}}+{\hat{\varepsilon }}_{\mathrm{frac}}$$

Finally, frequency offset compensation is performed in the time domain on the resampled received sequence:

(23) $$r_{\mathrm{cfo}}[n]=r[n]\exp\left(-j2\pi \frac{\hat{\varepsilon }n}{N_{\mathrm{FFT}}}\right)$$

Since the proposed transmitter uses optical heterodyne beating between two free-running ECLs and the receiver adopts homodyne down-conversion, the received baseband signal contains residual phase-related impairments originating from both the optical beat note and the electrical local oscillator. The phase of the generated D-band carrier is determined by the relative phase between the two optical tones. For two independent ECLs, the linewidth of the heterodyne beat note can be approximated by the sum of the two laser linewidths. In this experiment, both ECLs have linewidths below 100 kHz, and therefore the estimated upper bound of the RF beat linewidth is approximately 200 kHz.

For the 56-GBaud OFDM signal with 2048 FFT points, the subcarrier spacing is 27.34 MHz, while that of the 32-GBaud reference signal is 15.63 MHz. Accordingly, the estimated beat-linewidth-to-subcarrier-spacing ratios are below 7.3 × 10^−3^ and 1.28 × 10^−2^, respectively. These values are much smaller than one subcarrier spacing, indicating that the dominant phase-related impairment can be treated as slow carrier-frequency offset and common phase error rather than severe fast inter-carrier interference. This consideration supports the use of the two-stage frequency offset compensation procedure described above.

### 3.4. Channel Estimation, Equalizer Configuration, and Phase Recovery

After frequency offset compensation, the CP and CS samples are removed, and a 2048-point FFT is performed on each effective OFDM symbol. Let the frequency-domain received symbol of the mth OFDM symbol on the kth subcarrier be $Y_{m}\left[k\right]$.

The initial channel response is estimated from the training OFDM symbol using the LS method:

(24) $${\hat{H}}_{0}[k]=\frac{Y_{0}[k]}{X_{0}[k]},\;k\in A$$

where $X_{0}\left[k\right]$ and $Y_{0}\left[k\right]$ denote the transmitted and received training symbols on the kth active subcarrier, respectively. For subsequent data OFDM symbols, pilot subcarriers are used to update the channel response. On the pilot-subcarrier set P, the pilot-based LS channel estimate is

(25) $${\hat{H}}_{m}[p]=\frac{Y_{m}[p]}{X_{m}[p]},\;p\in P,\;m=1,\ldots ,5$$

where $X_{m}\left[p\right]$ is the known transmitted pilot on the pth pilot subcarrier. The channel estimates on all active subcarriers are then obtained by linear interpolation:

(26) $${\hat{H}}_{m}[k]=\mathrm{Interp}\left\{{\hat{H}}_{m}[p],p\in P\right\},\;k\in A$$

Based on the estimated channel response, one-tap zero-forcing frequency-domain equalization is performed on each active subcarrier:

(27) $$Z_{m}[k]=\frac{Y_{m}[k]}{{\hat{H}}_{m}[k]},\;k\in A$$

This equalizer can compensate the deterministic amplitude and phase responses of the end-to-end link on a per-subcarrier basis. However, when bandwidth roll-off causes a decrease in the received SNR at the edge subcarriers, the receiver-side one-tap equalizer cannot recover this SNR loss. Consequently, the wider-bandwidth 56-GBaud OFDM signal exhibits more pronounced performance degradation at the edge subcarriers, which is consistent with the experimental results in Section 4.

After channel equalization, the pilot subcarriers are further used to estimate the residual CPE of each data OFDM symbol. The phase-rotation estimate for the mth data OFDM symbol is

(28) $${\hat{\phi }}_{m}=\arg\left[\sum_{p\in P}Z_{m}[p]\mathrm{conj}\left(X_{m}[p]\right)\right]$$

The equalized subcarriers are then phase-corrected as

(29) $$X_{m}[k]=Z_{m}[k]\exp\left(-j{\hat{\phi }}_{m}\right),\;k\in A$$

Finally, QPSK decision and BER statistics are performed on ${\tilde{X}}_{m}\left[k\right]$. This pilot-assisted phase-recovery procedure is executed once for each data OFDM symbol and can effectively compensate the slow common phase rotation caused by laser phase noise, local-oscillator drift, and residual frequency offset estimation errors. If the phase fluctuation varies rapidly within one OFDM symbol, it manifests as residual inter-carrier interference (ICI), which cannot be completely eliminated by one-tap equalization. In the current experiment, the measured phase response remains smooth and repeatable across multiple frames, indicating that rapid phase noise is not a primary limiting factor in this QPSK-OFDM transmission experiment.

After frequency offset compensation, residual common phase error is estimated from the pilot subcarriers and corrected on an OFDM-symbol basis. This pilot-assisted CPE correction is suitable for the present QPSK-OFDM experiment because the dominant phase fluctuation is slow relative to one OFDM symbol. Rapid intra-symbol phase fluctuation would appear as residual ICI, which cannot be fully removed by one-tap equalization.

### 3.5. Feasibility and Limitations of Nonlinear Compensation Techniques

Nonlinear compensation was not adopted in the current experiment. In principle, nonlinear impairments in a photonic-assisted D-band link can be mitigated by transmitter-side digital pre-distortion (DPD), receiver-side Volterra equalization, memory-polynomial equalization, or neural-network-based nonlinear equalization. If future systems can provide an accurate nonlinear model or a sufficiently long training sequence, these nonlinear compensation techniques can be further implemented.

For the current experimental platform, nonlinear compensation was not introduced in this paper mainly for three reasons. First, possible nonlinear distortion is distributed across multiple devices and link segments, including the electrical amplifier, IQ-MZM, UTC-PD, D-band mixer, local-oscillator chain, and baseband receiver front-end. Therefore, relying solely on the received OFDM data makes it difficult to separate and accurately model the nonlinear response of each device. Second, nonlinear equalization methods such as Volterra equalizers or memory-polynomial equalizers require additional training overhead, and the nonlinear order, memory length, and regularization parameters must be carefully selected. For OFDM waveforms with a high peak-to-average power ratio (PAPR), an insufficiently trained nonlinear equalizer may overfit the received data or amplify noise on low-SNR edge subcarriers. Third, the experimental results show that the dominant impairments of the current system are the frequency-selective amplitude and phase responses caused by the bandwidth-limited link, whereas only slight nonlinear degradation is observed at high optical power.

For these reasons, the current receiver-side DSP mainly adopts linear pilot-assisted one-tap frequency-domain equalization and per-OFDM-symbol CPE correction. Meanwhile, by optimizing the optical power before the UTC-PD, the experiment prevents the photoelectric conversion and subsequent analog front-end link from operating in a strongly nonlinear region. Future work will combine device-level calibration, feedback-path design, and training-sequence optimization to further investigate more advanced nonlinear compensation methods, including transmitter-side DPD, receiver-side Volterra equalization, and memory-polynomial equalization.

## 4. Experimental Results and Analysis

To evaluate the proposed 138 GHz D-band photonics-aided wireless transmission system with homodyne reception, the transmitted and received spectra, received constellations, BER performance under different transmitted optical powers, and channel amplitude/phase responses are analyzed in this section. Since the main objective of this work is to verify the feasibility of 56-GBaud OFDM-QPSK transmission over a 20 m indoor line-of-sight link, a 32-GBaud OFDM-QPSK signal is also included as a reference case under the same experimental platform, modulation format, and receiver-side DSP flow. This comparison helps clarify the influence of occupied bandwidth on transmission performance.

### 4.1. Analysis of Transmitter and Receiver Spectra

**Figure 3:**
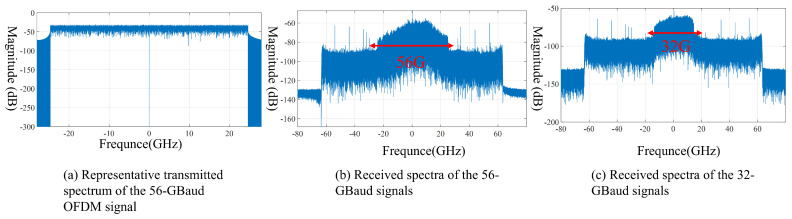
Spectra of the 56-GBaud and 32-GBaud OFDM signals.

Figure 3a shows a representative transmitted spectrum of the 56-GBaud OFDM signal. As expected for the adopted OFDM waveform, the signal energy is mainly concentrated within the effective occupied bandwidth, and the spectrum is approximately symmetric with respect to the center frequency. A reserved null band is visible around the center, which is beneficial for suppressing the influence of DC components and local-oscillator leakage in the homodyne receiver.

Figure 3b,c present the received spectra of the 56-GBaud and 32-GBaud OFDM signals, respectively, after 20 m indoor wireless transmission and homodyne down-conversion. In both cases, the overall spectral envelope is well preserved and continuous over the occupied band, indicating that the proposed transmitter, wireless link, and receiver can support broadband signal generation, transmission, and recovery at 138 GHz. This result directly confirms the feasibility of the photonics-aided D-band architecture for high-speed indoor wireless delivery.

At the same time, the received spectra are not completely flat. Noticeable passband fluctuations and gradual attenuation toward the band edges can be observed, revealing that the end-to-end link exhibits frequency-selective characteristics. This frequency selectivity has a stronger impact on the 56-GBaud signal because its wider occupied bandwidth causes more subcarriers to extend into the less favorable edge region of the link response. In contrast, the 32-GBaud signal occupies a narrower bandwidth and is therefore more concentrated within the relatively flat central region. This difference already suggests that the 32-GBaud signal should exhibit better robustness after equalization, which is further confirmed by the constellation and BER results discussed below.

### 4.2. Analysis of Received Constellation Diagrams

**Figure 4:**
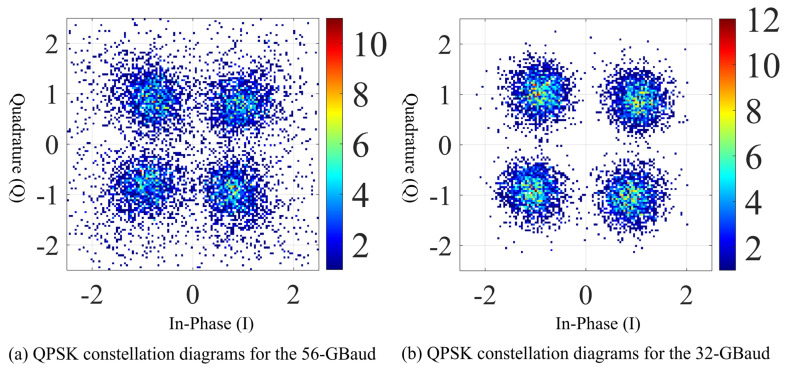
QPSK constellation diagrams for the 56-GBaud and 32-GBaud signals.

Figure 4 shows the received QPSK constellation diagrams for the 56-GBaud and 32-GBaud OFDM signals after receiver-side DSP. In both cases, four clearly distinguishable constellation clusters are obtained, indicating that the adopted DSP chain can effectively accomplish symbol synchronization, frequency offset compensation, channel estimation, equalization, and residual phase correction. Therefore, the proposed homodyne receiver combined with offline DSP is capable of reliably recovering the OFDM baseband data after 20 m D-band wireless transmission.

A comparison between the two constellations shows that the 32-GBaud signal exhibits more compact clustering, while the 56-GBaud signal still presents a certain degree of dispersion. This difference is consistent with the spectral observations in Figure 3. Because the 56-GBaud signal occupies a wider bandwidth, a larger fraction of its subcarriers is affected by the non-flat amplitude and phase response of the link, especially near the band edges. As a result, although pilot-aided equalization and phase compensation can remove a substantial part of the distortion, some residual impairment remains in the recovered constellation.

It is worth noting that the 56-GBaud constellation still maintains clear quadrant separation and good symbol distinguishability. This means that the degradation is mainly reflected in a larger distribution spread rather than a complete loss of symbol decision boundaries. In other words, the experimental results show that the present system is already capable of supporting stable 56-GBaud OFDM-QPSK transmission, while the remaining performance penalty is mainly associated with bandwidth-related link distortion rather than failure of synchronization or carrier recovery.

**Figure 5:**
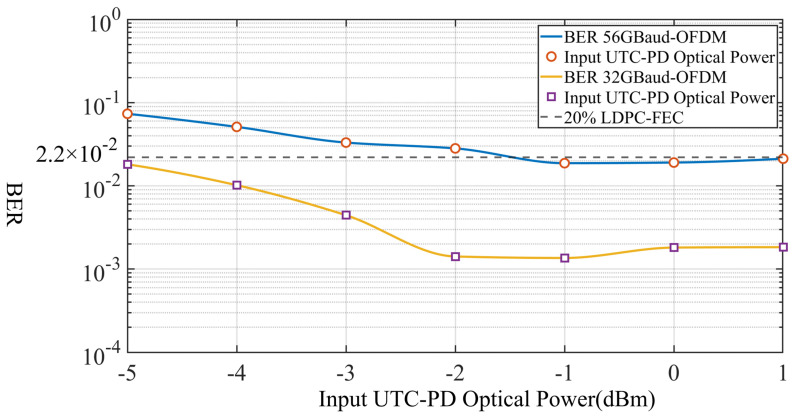
BER curves for 56-GBaud and 32-GBaud OFDM-QPSK signals across various levels of input optical power.

Figure 5 shows the BER performance of the 56-GBaud and 32-GBaud OFDM-QPSK signals as a function of transmitted optical power before the PD. For both symbol rates, the BER first decreases as the transmitted optical power increases, then reaches a relatively favorable region, and finally exhibits a slight degradation at higher power. This trend indicates that the system has an optimal operating optical-power range, approximately between −2 dBm and −1 dBm.

In the low-power region, the BER is relatively high because the received signal strength is insufficient, resulting in a limited effective signal-to-noise ratio after down-conversion and sampling. As the transmitted optical power increases, the recovered signal quality improves and the BER decreases correspondingly. However, when the power continues to increase beyond the optimal range, the BER no longer improves and even shows a slight increase. This behavior suggests that, under high-power conditions, the system may begin to experience slight nonlinear effects in the optical-to-electrical conversion or subsequent analog front-end stages, which is consistent with the overall interpretation given in this work.

Across the entire tested power range, the 32-GBaud signal consistently achieves better BER performance than the 56-GBaud signal. Since the two cases use the same hardware platform, modulation format, and DSP flow, this performance gap can be mainly attributed to the difference in occupied bandwidth. The narrower 32-GBaud signal places most of its effective subcarriers within the flatter portion of the channel response, while the 56-GBaud signal extends further into the frequency-selective edge region, making it more vulnerable to amplitude roll-off and phase distortion. Even so, the 56-GBaud signal can still approach the 20% LDPC-FEC threshold at a transmitted optical power of approximately −1 dBm, which verifies the feasibility of high-symbol-rate D-band wireless transmission in the proposed system.

### 4.3. Analysis of Channel Amplitude and Phase Responses

**Figure 6:**
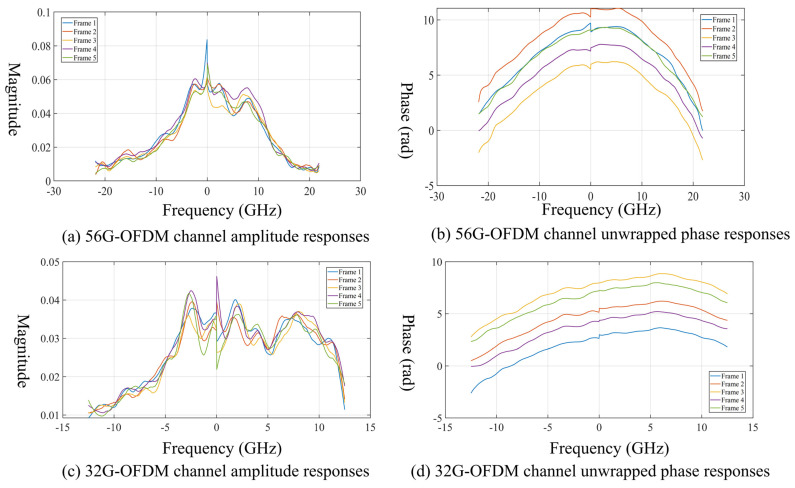
Estimated channel amplitude and unwrapped phase responses over five received frames for the 56-GBaud and 32-GBaud OFDM signals.

Figure 6 further presents the channel amplitude and phase responses for five frames of received data. Figure 6a,b correspond to the 56-GBaud signal, while Figure 6c,d correspond to the 32-GBaud signal. For both symbol rates, the overall shapes of the amplitude and phase curves remain similar across different frames. This indicates that the 20 m indoor D-band wireless link has good short-term stability over the measurement interval and that the link response is sufficiently repeatable for training- and pilot-assisted DSP.

From the amplitude-response results, it can be clearly seen that the channel response is relatively higher in the middle part of the occupied band and gradually rolls off toward the edges. This confirms that the link is frequency selective rather than ideally flat. Such selectivity is an important reason for the performance difference between the two symbol rates. Because the 56-GBaud signal spans a wider frequency range, more of its active subcarriers fall into the distorted edge region, where the response deviates further from the ideal condition. By contrast, the 32-GBaud signal is more concentrated in the central region with a comparatively flatter channel amplitude response, which is favorable for signal recovery.

The measured phase responses in Figure 6 further verify the above DSP assumption. For both the 56-GBaud and 32-GBaud signals, the unwrapped phase responses remain smooth and repeatable over five received frames. In the experiment, the estimated normalized frequency offsets are approximately 15.9947 and 31.0005 for the 56-GBaud and 32-GBaud signals, corresponding to frequency offsets of 437.36 MHz and 484.38 MHz, respectively. After the two-stage frequency offset compensation and pilot-assisted CPE correction, no rapidly varying random phase fluctuation is observed in the recovered channel responses. Therefore, the dominant phase-related impairments in the current system are mainly slow carrier-frequency offset, common phase error, and quasi-static channel phase distortion, all of which can be effectively handled by the adopted DSP chain.

Consequently, the remaining performance difference between the 56-GBaud and 32-GBaud signals is mainly attributed to the bandwidth-constrained frequency-selective amplitude and phase responses of the end-to-end D-band link. The wider 56-GBaud signal occupies more edge subcarriers, where the link response is more strongly attenuated and distorted. Although one-tap equalization can compensate deterministic amplitude and phase distortion, it cannot recover the SNR loss of the attenuated edge subcarriers. This explains why the 32-GBaud signal exhibits better BER and constellation performance under the same hardware platform.

These results indicate that phase-related impairments are not the primary limiting factor in the present QPSK-OFDM experiment; instead, the system performance is mainly constrained by frequency-selective amplitude/phase distortion and the associated edge-subcarrier SNR degradation.

## 5. Conclusions

In this paper, we experimentally demonstrated a photonics-aided 138-GHz D-band wireless transmission system using optical heterodyne generation and homodyne down-conversion. A 56-GBaud OFDM-QPSK signal was successfully transmitted over a 20 m indoor line-of-sight wireless link, achieving a net bit rate of 81.16 Gbit/s under the adopted OFDM overhead configuration. The experimental results, including measured spectra, recovered constellations, BER curves, and channel responses, verify the feasibility of high-baud-rate D-band wireless transmission based on a photonics-aided homodyne architecture.

The performance analysis shows that the main limitation of the 56-GBaud signal is the frequency-selective amplitude roll-off of the end-to-end transmitter and receiver response. Compared with the 32-GBaud reference signal, the 56-GBaud signal occupies a wider bandwidth and is therefore more strongly affected by the non-flat response of the D-band hardware chain. Although the receiver DSP can compensate for the slow carrier-frequency offset, common phase rotation, and part of the linear channel distortion, the SNR loss of the attenuated edge subcarriers cannot be fully recovered by receiver-side equalization alone.

In particular, the combined ECL linewidth is much smaller than the OFDM subcarrier spacing, and the measured normalized frequency offset and smooth frame-to-frame phase responses confirm that the dominant phase-related impairments can be treated as slow carrier-frequency offset and common phase error. This explains why the adopted DSP chain is sufficient for QPSK-OFDM recovery in the current experiment, although further laser/LO stabilization would be required for higher-order QAM or stricter EVM requirements.

The results indicate that photonics-aided D-band homodyne transmission is a promising candidate for short-range high-capacity indoor wireless access and future 6G fiber–wireless integration. Future work will focus on phase-noise reduction, laser and LO stabilization, transmitter-side pre-equalization or digital pre-distortion, flatter D-band front-end design, higher-order QAM transmission, and systematic evaluation of antenna alignment tolerance and environmental robustness.

## Figures and Tables

**Table 1:** Representative OFDM wireless transmission demonstrations at the D-band and above reported in the last decade.

Year	Reference	Carrier Frequency	OFDM Format/Bandwidth or Baud Rate	Rate Metric	Wireless Distance	RX Architecture
2020	[19]	350 GHz	PS-16QAM-OFDM, 33 GHz total bandwidth	106.2 Gbit/s over 10 km fiber + 26.8 m wireless; 119.1 Gbit/s direct wireless	26.8 m	Schottky sub-harmonic mixer, 40-GHz IF
2021	[25]	300 GHz	5G-based OFDM; 8 × 100 MHz component carriers	1.68 Gbit/s real-time video transfer (OFDM PoC)	10 m	Self-heterodyne; BB unit generates OFDM at 10.5-GHz IF
2022	[26]	D-band	OFDM 16QAM/PS-64QAM	40/55 Gbit/s	200 m	Not stated in abstract
2023	[34]	320 GHz	23-GBaud 16-QAM OFDM, 2 × 2 MIMO	60.5 Gbit/s net	3 m (+20 km fiber)	Not stated in abstract
2025	[35]	339 GHz	PS-64QAM-OFDM	37.04 Gbit/s	100 m	Not stated in abstract
2026	This work	138 GHz	56-GBaud OFDM-QPSK	81.16 Gbit/s	20 m	Homodyne/zero-IF, direct down-conversion to baseband

**Table 2:** OFDM signal parameters.

Parameter	Value
FFT Points	2048
Effective Subcarriers	1600
Pilot Subcarriers	202
Pilot Insertion Method	1 pilot inserted every 8 subcarriers
Cyclic Prefix Length (CP)	128
Cyclic Suffix Length (CS)	32
Frame Structure	1 OFDM training symbol, 5 OFDM data symbols
Modulation Format	QPSK
System Type	OFDM

**Table 3:** Device parameters.

Component	Parameter
ECL1	Output: 1550 nm@10 dBm Linewidth: <100 kHz
ECL2	Output: 1551.1 nm@10 dBm Linewidth: <100 kHz
PM-OC	Operating wavelength: 1530~1563 nm
UTC-PD	Typical operating frequency: 110–170 GHz, Typical output power: −7 dBm
HA	3 dB beamwidth: ~10°, Gain: 25 dB gain, Typical operating frequency: 110–170 GHz
LO Mixer	Frequency: 138 GHz Conversion Loss: −15 dB, Imaging Rejection: −15 dBc
AWG	The rate of sampling: 224 GSa/s
DSO	The rate of sampling: 160 GSa/s, Electrical bandwidth: 62 GHz

## Data Availability

The raw data supporting the conclusions of this article will be made available by the authors on request.
